# A standardised bioassay method using a bench‐top spray tower to evaluate entomopathogenic fungi for control of the greenhouse whitefly, *Trialeurodes vaporariorum*

**DOI:** 10.1002/ps.5794

**Published:** 2020-03-12

**Authors:** Eleanor L Spence, David Chandler, Steve Edgington, Shaun D Berry, Gareth Martin, Christine O'Sullivan, Claus Svendsen, Helen Hesketh

**Affiliations:** ^1^ UK Centre for Ecology & Hydrology, Benson Lane Crowmarsh Gifford Wallingford UK; ^2^ Warwick Crop Centre, School of Life Sciences, Wellesbourne Campus The University of Warwick Warwick UK; ^3^ CABI Bakeham Lane Egham UK; ^4^ BASF Corporation Research Triangle Park; ^5^ BASF plc Woolpit UK; ^6^ Silsoe Spray Applications Unit Ltd Bedfordshire UK

**Keywords:** entomopathogenic fungus, spray tower, bioassay, biopesticide, whitefly

## Abstract

**BACKGROUND:**

Bioassays evaluating entomopathogenic fungi (EPF) isolates for effective microbial control of whitefly are a fundamental part of the screening process for bioprotectants, but development of repeatable, robust bioassays is not straightforward. Currently, there is no readily available standardised method to test the efficacy of EPF on whitefly.

Here, we describe the calibration and use of a spray tower to deliver a standardised protocol to assess EPF activity; the method was validated using 18 EPF from four genera in tests against greenhouse whitefly, *Trialeurodes vaporariorum* (Westwood*)*.

**RESULTS:**

At 138 kPa, the sprayer delivered 0.062 mL mm^−2^ (620 L ha^−1^) and an even deposition of spray across the central 1590 mm^2^ of the spray area.

Average conidial deposition for all EPF was 252 conidia mm^−2^ and equivalent to 2.5 × 10^12^ conidia ha^−1^ at an application concentration of 1 × 10^7^ conidia mL^−1^. Conidial deposition of a test *Beauveria bassiana* suspension increased with increasing application concentration.

Egg laying by *T. vaporariorum* adults was restricted to 177 mm^2^ using clip cages specifically designed to ensure that third‐instar *T. vaporariorum* received a uniform spray coverage. Nymphs occupied 373 ± 5 mm^2^ of the leaf after migrating during the first instar.

Average *T. vaporariorum* mortality totaled 8–89% 14 days after application of 1 × 10^7^ conidia mL^−1^ of each EPF isolate.

**CONCLUSION:**

Combining the calibrated sprayer and bioassay method provides a reliable, standardised approach to test the virulence of EPF against whitefly nymphs. This laboratory‐based assay is affordable, replicable and allows the user to alter the dose of conidia applied to the target.

## Graphical abstract

Entomopathogenic fungi (EPF) are commercially developed as microbial bioprotectants for whitefly control. Here, we describe the calibration and use of a pressurised spray tower to deliver a standardised laboratory protocol to allow 14 day assessments of EPF activity; the method was validated using 18 EPF from four genera in tests against greenhouse whitefly, *Trialeurodes vaporariorum*. This assay is affordable, replicable and allows the user to alter the dose of spores applied to the target area.

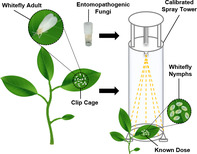

© 2020 The Authors. *Pest Management Science* published by John Wiley & Sons Ltd on behalf of Society of Chemical Industry.

## 1 INTRODUCTION

The greenhouse whitefly (*Trialeurodes vaporariorum* Westwood, Hemiptera: Aleyrodidae) is a highly polyphagous homopteran pest of more than 300 host plant species^1^ and causes extensive pest damage to crops globally. The species originates from tropical or subtropical America,^2^ but has become established in all continents except Antarctica.^3^ Direct plant damage is caused by adults and nymphs feeding on phloem, while indirect damage occurs through secretion of honeydew, which supports growth of sooty moulds (*Cladosphaerospermum* spp.) that reduce plant photosynthesis, leading to stunted growth.^4^ However, the main problems occur through transmission of *Criniviruses* (positive sense single‐stranded RNA viruses vectored exclusively by whitefly).^5^ Crop damage caused by *T. vaporariorum* vectored viruses is estimated to cost the global agricultural economy more than US$1 billion a year.^6^, ^7^, ^8^

Management of *T. vaporariorum* using synthetic chemical pesticides is made difficult because of widespread pesticide resistance. Resistance has been documented to all major pesticide groups, including pyrethroids, organophosphates, carbamates and neonicotinoids, as well as the insect growth regulator buprofezin.^9^, ^10^, ^11^, ^12^ Biological control of *T. vaporariorum* has been used successfully by growers of protected crops in the UK and the Netherlands for over 40 years.^9^ The most popular approach is the inundative application of parasitoids (mainly *Encarsia formosa* Gahan, Hymenoptera: Aphelinidae and *Eretmocerus eremicus* Rose and Zolnerowich, Hymenoptera: Aphelinidae) and predators (*Amblyseius swirskii* Athias‐Henriot, Acari: Phytoseiidae, *Transeius montdorensis* Schicha, Acari: Phytoseiidae, *Delphastus catalinae* Horn, Coleoptera: Coccinellidae and *Macrolophus pygmaeus* Rambur, Hemiptera: Miridae).^9^, ^13^ There are occasions when whitefly populations can outstrip the ability of predators and parasitoids to control them, and under these circumstances conventional pesticides are applied as a supplemental treatment. However, given the problems with pesticide resistance, there is a need for alternative interventions as part of a more sustainable, integrated pest management (IPM) approach. Biopesticides (increasingly referred to as bioprotectants) are microorganisms, plant extracts or semio‐chemicals that are used against pests in plant protection.^14^ In 2018, biopesticides represented 18% of the US$56 billion pesticide market, with a compound annual growth rate of more than 16% (www.marketsandmarkets.com). The number of registered biopesticides in Europe is increasing, largely due to legislation introduced by the EU Sustainable Use Directive on pesticides (2009/128/EC), which aims to prioritise non‐chemical alternatives over chemical control of insect pests as part of IPM.^15^ The development of biopesticides costs significantly less than synthetic pesticides, encouraging agrochemical companies to expand their range of biological products.^16^

The most effective microbial pathogens against *T. vaporariorum* are entomopathogenic fungi (insect killing fungi; EPF) which infect the insect through direct penetration of the cuticle.^17^ Bacteria and viruses are not considered an option as they must be ingested by whitefly (whose mouthparts remain inside host plant tissues whilst they feed) although rare infections through existing wounds can sometimes occur.^13^ Female *T. vaporariorum* lay eggs on the lower surface of leaves and on hatching, first instars ‘crawl’ until they find a suitable location to obtain phloem.^18^ Nymphs then remain in this feeding location until they emerge as adults. The most susceptible stages of *T. vaporariorum* to EPF are the first, second and third instars^19^ but due to their immobility, nymphs will only become infected by conidia that are sprayed directly onto their cuticle, and secondary acquisition of conidia only occurs by the adult walking across the plant surface.^20^ The development of EPF as bioprotectants, or ‘mycoinsecticides’, has been successful in greenhouse, horticultural, orchard and arable field crops.^21^, ^22^, ^23^, ^24^, ^25^ The most common approach for whitefly control using EPF involves the inundative application of large numbers of infective conidia^26^; indeed, many anamorphic (= asexual) EPF from the order Hypocreales are easily mass reared on culture media and an increasing number have been developed as proprietary bioprotectants against a range of pest species.^27^ The risk of resistance developing to EPF is reported to be very low,^28^, ^29^ there are little to no residues left on the crop and EPF are often compatible with other natural enemies.^30^ EPF can be successfully used in IPM as a preventative measure as a second line of defence to supplement the use of predators and parasitoids. Several EPF are commercially available in Europe to target *T. vaporariorum*, such as *Beauveria bassiana* (Balsamo) (Hypocreales: Clavicipitaceae) (Naturalis®, Intrachem Bio Italia; Botanigard®, Certis), *Cordyceps* (=*Isaria*) *fumosorosea* (Wize) (Hypocreales: Cordycipitaceae) (PreFeRal®, SePRO Corporation; Nofly, Natural Industries, Inc.) and *Akanthomyces* (=*Lecanicillium*) *muscarius* (Petch) (Hypocreales: Cordycipitaceae) (Mycotal®, Koppert Biological systems).

Determining the pathogenicity of EPF to demonstrate the potential to control *T. vaporariorum* is a fundamentally important step in the development of EPF as a biological control option, particularly at the screening stages. To ensure consistent results, testing potential biological insecticides in the laboratory requires a method to deliver reproducible doses of test substances. There are two approaches commonly used to apply a known concentration of an EPF to *T. vaporariorum* nymphs: dipping the target in a suspension for a known length of time^31^ or spraying the target with a known volume of suspension.^32^, ^33^ Dipping target leaves in a fungal suspension provides 100% contact between *T. vaporariorum* and the EPF conidia but it is difficult to provide a reproducible dose because of run‐off from the target. Spray applications in the laboratory are often performed using an air‐assisted Potter tower or similar spraying equipment to provide uniform coverage of the target area^34^, ^35^, ^36^, ^37^, ^38^ and whilst this is highly efficient, the equipment is often expensive and therefore inaccessible to some researchers.

A cheap alternative sprayer for laboratory work has been suggested by Mascarin *et al*.^20^, ^39^ based on a portable artist airbrush that could readily be used for testing chemical toxicity and EPF effectiveness. This has subsequently been used to determine the virulence of EPF against several pests, including the silverleaf whitefly *Bemisia tabaci* (Gennadius) (Hemiptera: Aleyrodidae) and the carmine spider mite *Tetranychus cinnabarinus* (Boisduval) **(**Acari: Tetranychidae**)** 20, 40 in detached leaf bioassays. Here we report on the development of a standardised and repeatable bioassay method using this sprayer device which we used to assess the pathogenicity of a number of EPF to *T. vaporariorum*. We describe an integrated bioassay approach, including a clip cage (designed as an important component of the bioassay set‐up) which when used alongside the sprayer provides reproducible dose concentrations to a known target area in whole‐plant assays. This bioassay approach was validated by testing the pathogenicity of 18 EPF isolates in whole‐plant assays from four genera of hypocrealean fungi (*Beauveria*, *Cordyceps*, *Akanthomyces* and *Metarhizium*) to third‐instar *T. vaporariorum*.

## 2 METHODS

### 2.1 Insect, plant and fungal cultures

A stock culture of *T. vaporariorum* was obtained from a colony held at the University of Warwick (UK) which originated from a natural population found in Evesham UK in 2018. Stock *T. vaporariorum* were subsequently maintained on small aubergine plants (*Solanum melongena* L., Polemoniales:Solanaceae, var. Paris; Ramiro Arnedo, Spain) in 60 × 60 × 60 cm nylon and Perspex insect‐rearing tents (BugDorm‐2® insect rearing tent, Watkins & Doncaster, Pudleston, Leominster, UK). Every 14 days, fresh uninfested plants were added to the rearing tents and dead plants were removed. The *T. vaporariorum* cultures were maintained at 24 ± 0.5 °C under a light:dark 16:8 h photoperiod and all experiments were conducted under the same environmental conditions.

Aubergine seedlings used for bioassays were established from seeds sown individually in small plastic pots (4.5 × 4.5 × 5.5 cm) in 50 g of compost (John Innes Seed and Cutting compost) and were kept pesticide free. Plants were grown in a greenhouse under a light:dark 16:8 h photoperiod with supplemented overhead lighting to ensure a minimum light intensity of 300 μmol m^–2^ s^–1^. The temperature in the greenhouse was maintained at 25 ± 3 °C during daylight hours and 15 ± 2 °C at night. Seedlings were approximately 7 weeks old and at the two to three true leaf stage when they were used in bioassays.

Fungal isolates were obtained from several sources (see Table 1). The United States Department of Agriculture Agricultural Research Service collection of entomopathogenic fungal cultures (ARSEF database: https://data.nal.usda.gov) supplied 11 isolates. Five isolates were from the University of Warwick and a commercial sample of *B. bassiana* isolate PPRI5339 was supplied by BASF plc. The remaining isolate was provided from the Centre for Ecology & Hydrology (Wallingford, UK) entomopathogenic fungus culture collection. Isolates were selected from origins of varied climates and were originally isolated from a number of different host species, including *Bemisia tabaci*, *T. vaporariorum* and Hemiptera: Aphididae. Re‐isolations of fungi purchased from commercially available biological control products were also used (see Table 1). Samples from the ARSEF database were spread onto 10 mL of Sabouraud Dextrose Agar (SDA; 65 g per 1 L deionised water) in 9 cm plastic triple‐vented Petri dishes. Fungal isolates from commercial products were re‐isolated by streak spreading the product pellets/powder across SDA in Petri dishes using sterile plastic spreaders. All Petri dishes were sealed with Parafilm and then incubated in the dark at 25 °C for 1 week. After this incubation period, three plugs were taken from each Petri dish using a sterile ‘cork’ borer (diameter 7 mm) and stored in 1 mL of aqueous glycerol (10% by volume). The fungal isolates were stored in cryovials (1.2 mL, Thermo Scientific Nalgene) at −80 °C as first or second subcultures to prevent attenuation of virulence through repeated subculturing.

**Table 1:** Identification of EPF used in bioassays against third instar greenhouse whitefly (*Trialeurodes vaporariorum*)

ARSEF ID/name	Species	Strain	Host^a^	Origin^a^
ATCC 5278	*Beauveria bassiana*		*Bemisia tabaci*	Vermont, USA
ATCC 6920	*Beauveria bassiana*		*Trialeurodes vaporariorum*	Canada
ATCC 6921	*Beauveria bassiana*		*Trialeurodes vaporariorum*	Canada
ATCC 9451	*Beauveria bassiana*		*Trialeurodes vaporariorum*	Kazakhstan
Botanigard	*Beauveria bassiana*	GHA	N/A	Product
GHA	*Beauveria bassiana*	GHA	*Diabrotica undecimpunctata*	Oregon, USA
PPRI5339	*Beauveria bassiana*	PPRI5339	N/A	Product
ATCC 7477	*Cordyceps javanica*		*Trialeurodes vaporariorum*	Argentina
ATCC 4412	*Cordyceps farinosa*		*Trialeurodes vaporariorum*	Malaysia
ATCC 2658	*Cordyceps fumosorosea*		*Trialeurodes vaporariorum*	Florida, USA
PFR	*Cordyceps fumosorosea*	Apopka 97	Aphididae	USA
ATCC 4205	*Cordyceps fumosorosea*		*Trialeurodes vaporariorum*	Malaysia
ATCC 4060	*Akanthomyces lecanii*		*Trialeurodes vaporariorum*	Malaysia
ATCC 972	*Akanthomyces lecanii*		*Trialeurodes vaporariorum*	Poland
ATCC 6544	*Akanthomyces lecanii*		*Trialeurodes vaporariorum*	UK
Vertalec	*Akanthomyces muscarius*	Ve6	N/A	Product
Met 52	*Metarhizium brunneum*	F52	N/A	Product
Bioblast	*Metarhizium anisopliae*	ESC1	N/A	Product

a Host and origin: information taken from the ARSEF database.

Fungal cultures for experiments were prepared by defrosting, mixing and spreading each test isolate onto 10 mL of SDA using a sterile plastic spreader. Plates were sealed with Parafilm and incubated at 25 °C for 14 days in the dark. To prepare conidia suspensions for bioassays, conidia were removed by agitating the surface of the dish using a sterile pestle and Tween 80 (0.03% v/v). The conidia were immediately suspended in 3 mL of Tween 80 0.03% in a 50 mL tube and agitated vigorously on a vortex mixer for 2–3 min. The suspension was then filtered through a sterile muslin cloth to remove mycelia and culture debris. The concentrations of the resultant stock conidia suspensions were estimated by counting in an Improved Brightline Neubauer haemocytometer (×400 magnification) and subsequently diluted in sterile 0.03% Tween 80 to give a final concentration of 1 × 10^7^ conidia mL^−1^. Conidia suspensions were kept at 4 °C in the dark until used in experiments.

### 2.2 Sprayer design

**Figure 1:**
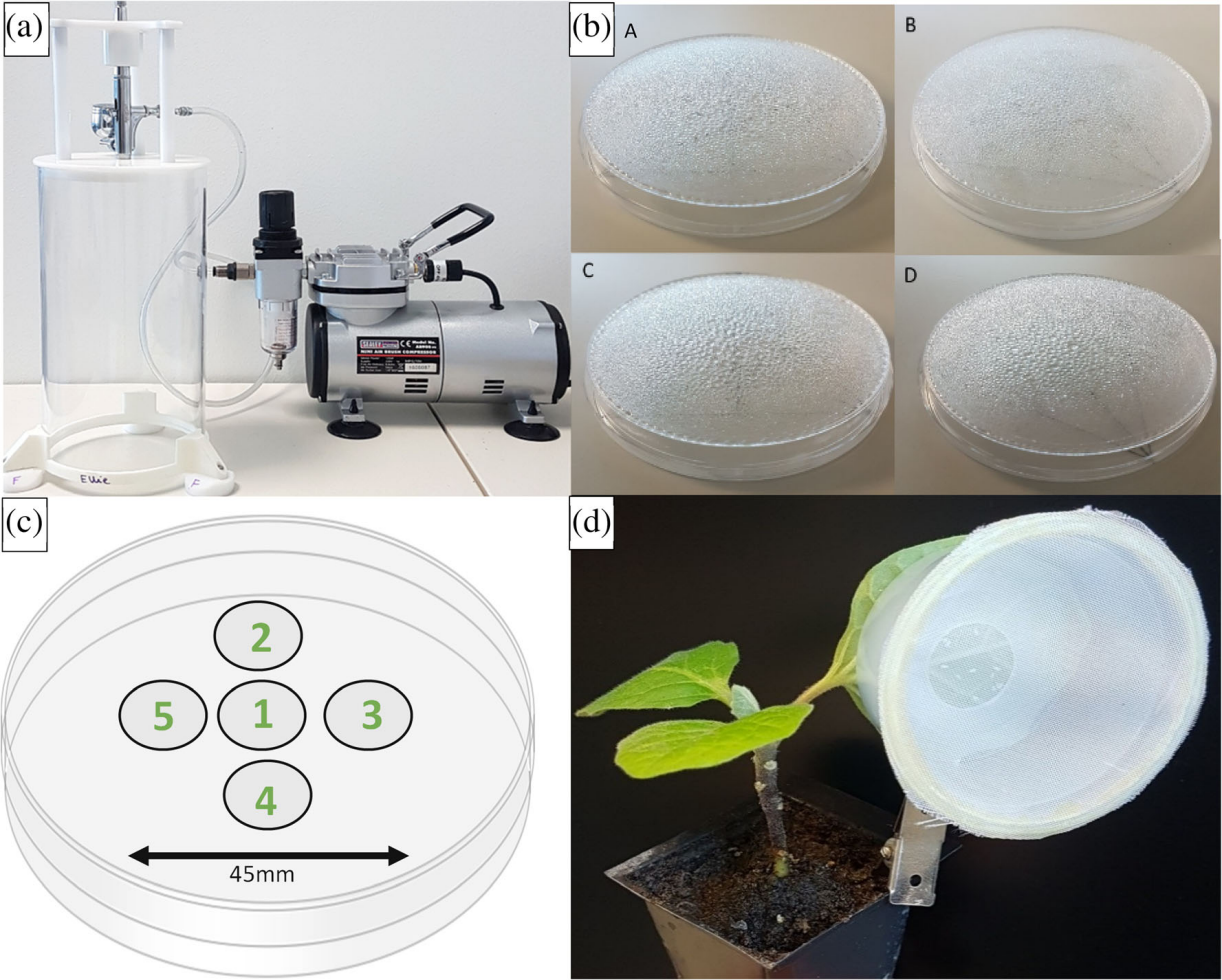
(a) The spray tower consisted of a gravity‐fed universal dual‐action airbrush (Spraycraft SP60) attached to a removable lid and acrylic cylinder (width 115 mm, height 240 mm). The spray tower was connected to a mini airbrush compressor (Sealey, Model no. AB900.V3). (b) Visible differences in water droplet size and variation across the surface of 90 mm diameter Petri dishes when sprayed with 1 mL of deionised water at (A) 103 kPa, (B) 138 kPa, (C) 172 kPa and (D) 207 kPa. (c) Arrangement of 13 mm diameter cover slips used to calibrate the volume of liquid deposited across the surface of a 90 mm diameter Petri dish. (d) Clip cages used to contain whitefly adults on a small known area of the abaxial leaf surface. Clip cages are made using truncated plastic pots, fine nylon mesh, acetate and metal hair clips.

The mini spray tower was built at the Centre of Ecology & Hydrology (Wallingford, UK) and was based on the design of Mascarin *et al*.^39^ with some modifications. A gravity‐fed universal dual action airbrush (Spraycraft SP60) was attached to a removable lid and placed on top of an acrylic cylinder (width 115 mm, height 240 mm) to form the spray tower. The cylinder was 3D printed and held 10 mm from the laboratory bench by removable feet (Fig. 1(a)). The airbrush sprayer was powered by a mini air brush compressor (Sealey, Model no. AB900.V3). All parts of the mini spray tower can be disassembled to be decontaminated. The sprayer is designed to fit a 90 mm Petri dish within the spray target area (base area 10,387 mm^2^).

### 2.3 Sprayer calibration

To calibrate the spray tower, the relationship between the volume of spray applied and the volume received in the target spray area at a range of spray pressures was investigated. A volume of 1 mL of deionised water was sprayed onto the base of an upturned empty 90 mm Petri dish at a range of pressures, namely 103.4, 137.9, 172.4 and 206.8 kPa. Five replicate sprays were applied to each of five separate dishes for each pressure and the weight of the Petri dish was measured before and after each spray. The size of water droplets across the Petri dish at each pressure was also compared by visual inspection (Fig. 1(b)).

Preliminary experiments indicated that the spray reaching the outer edge of the Petri dish was not uniform. Therefore, potential differential deposition of spray across the centre and toward the edges of the target spray area was investigated by spraying five 13 mm diameter circular glass cover slips, arranged across 45 mm in the formation shown in Fig. 1(c), to cover the whole target area (1590 mm^2^). A 1 mL sample of 0.1% stock solution (0.5 g/500 mL) of green dye (FastColours, E142 Green S) was sprayed onto the coverslips at 103.4, 137.9, 172.4 and 206.8 kPa and left to dry for 2 h. Coverslips were then individually submerged in 2 mL of deionised water in a 50 mL tube (Falcon 50 mL conical centrifuge tube, Fischer Scientific) and agitated on a vortex mixer for 2 minutes. A 200 μL aliquot was taken from each coverslip wash and loaded into a 96‐well plate (Nunclon Delta 96‐well Microwell plates, Thermo Scientific) and light absorbency at 634 nm of the solution was determined using a spectrophotometer (BioTek, Cytation 5). Each treatment had three replicate sprays (i.e. 15 coverslips per treatment) and the whole experiment was repeated three times to give a total of 45 test solutions at each of the three spray pressures. A standard calibration curve was created by measuring light absorbency for known concentrations of green dye which had been sprayed onto coverslips using the spray tower and this was used to estimate the concentration of green dye on each cover slip.

Tests were also performed to calibrate the sprayer for conidial deposition on the target area when an EPF was applied using the methods described above. Conidia suspensions of a representative EPF, *B. bassiana* (PPRI 5339), were prepared in sterile 0.03% Tween 80 at 10‐fold dilutions to give a range of concentrations from 1 × 10^4^ to 1 × 10^9^ conidia mL^−1^. Individual 22 × 22 mm square glass cover slips were placed in the centre of 90 mm diameter plastic Petri dishes and sprayed at 138 kPa, based on analysis of the previous results, with 1 mL of conidia suspension. Each concentration of suspension was sprayed onto three replicate cover slips and the whole experiment was repeated twice. Sprayed coverslips were immediately placed individually in 1 mL of 0.03% Tween 80 in 50 mL tubes and agitated on a vortex mixer for 2 min to dislodge conidia into suspension. Serial dilutions were made from 40 μL aliquots taken from each suspension, ensuring that each sample contained a low enough number of conidia for enumeration. Diluted suspensions were spread evenly across individual Petri dishes (90 mm diameter) containing 10 mL of SDA to give a total of 36 Petri dishes in the experiment. Dishes were sealed with Parafilm and incubated in the dark at 25 °C for 5 days. After that time, the number of colony‐forming units (CFUs) were counted and used to calculate the number of conidia received per square millimetre on each coverslip. By allowing conidia to germinate, this calibration method ensures that only infective viable conidia applied to the target area are enumerated.

### 2.4 Bioassay methods

Based on results from the experiments performed to calibrate the sprayer, it was ascertained that target *T. vaporariorum* nymphs needed to be restricted to a central target area of 1590 mm^2^ in order to receive a uniform dose of test chemical or fungal suspension from the spray tower. To achieve this, a method was developed to ensure that nymphs were constrained to the centre of leaves for spraying; clip cages (similar to those used to confine aphids individually to plant leaves^41^) were designed using truncated plastic pots (base diameter 40 mm and top diameter 50 mm; 25 mL frosted container from Ashwood, Derby, UK; Fig. 1(d)). A 15 mm diameter circle was cut into the centre of the base of each plastic pot to allow a limited area of the leaf to be exposed for *T. vaporariorum* adults to oviposit, i.e. the central target area identified in sprayer calibration experiments. The top of the pot was covered with fine nylon mesh (0.5 × 0.5 mm) and secured using hot glue. Metal hair clips were bent and glued to the side of each pot and to 30 mm diameter plastic acetate discs (0.3 mm thickness). The acetate disc held the cage flat to the ventral leaf surface and ensured the cage was as lightweight as possible to minimise damage to leaf surface tissues.

One clip cage containing 10–20 male and female adult *T. vaporariorum* was attached to the youngest available leaf. *Trialeurodes vaporariorum* adults were restricted to the abaxial leaf surface and exposed to a 15 mm diameter circle (area 177 mm^2^) of the leaf tissue. Individual plants were contained within ventilated 0.9 L transparent plastic pots (height 14.3 cm; width (rim, base) 9.4 cm, 6.7 cm, with a circle of nylon mesh for ventilation added to the lid; diameter 3.4 cm). Pots were maintained for 14 h at 24 °C under a 16:8 h light:dark photoperiod and after this period the adult *T. vaporariorum* were removed using a hand‐held aspirator. Eggs laid by *T. vaporariorum* adults were left to develop on individual plants *in situ* inside the ventilated plastic pots for 16 days until they reached the third instar. At this stage, the number of nymphs produced from the cohort of adults was counted and the distance between the most dispersed nymphs was measured using a digital calliper measuring tool (0–150 mm; Whitworth Precision) on both the horizontal and vertical axes. The approximate area that the nymphs occupied after migration was calculated by width × height.

The fungal pathogenicity of 18 different isolates (Table 1) was assessed in bioassays using third‐instar *T. vaporariorum*. Isolates were divided into three groups containing different isolates which were used as treatments in bioassays with experiments conducted 3 days apart in a randomised block design. Applications of the *B. bassiana* isolate PPRI5339 and the negative control of 0.03% Tween 80 (untreated nymphs) were replicated in every group as a standard for comparison. A single leaf per plant was infested with nymphs as described above and three replicate separate plants were used in each treatment. Due to variability between the numbers of eggs laid, this resulted in a range of between 51 and 146 nymphs per treatment. The number of nymphs was counted on each leaf prior to assays and assigned to treatments to evenly distribute the numbers of nymphs overall within a treatment and reduce variability as much as possible.

One millilitre of 1 × 10^7^ conidia mL^−1^ suspension was sprayed onto the third‐instar *T. vaporariorum* on the abaxial leaf surface using the spray tower at 138 kPa. The spray tower was cleaned between each spray by running ethanol (95%) through the artist spray gun, followed by Tween 80. The spray tower cylinder was cleaned using surface disinfectant (Rely^+^On Virkon) and wiped dry. Following each spray treatment, plants were left on the laboratory bench for 1–2 h until all leaves were dry. Following this, plants were placed inside 0.9 L plastic cages (as before) with unventilated lids in water‐filled trays for 48 h to maintain a high humidity. After 48 h, the lids were replaced with ventilated lids, thereby reducing humidity in order to prevent the growth of mildew on plants during the course of the assay. Plants were maintained at 24 ± 1.5 °C under a 16:8 h light:dark photoperiod. Every 48 h, the instar, emergence and mortality of *T. vaporariorum* were recorded for a total of 14 days following spray applications. *T. vaporariorum* instar was determined by size, shape and the presence or absence of red eyes, which indicate the last stage of the final instar. Infected nymphs appeared pink, brown or white depending on the species of fungus applied, whereas dead nymphs resulting from handling or other unknown causes appeared brown and desiccated. Hyphal growth emerged from the infected cadavers several days after death.

Between each treatment spray application, 1 mL of conidia suspension was also sprayed onto a 20 × 20 mm square glass coverslip. After each spray, cover slips were immediately placed in 1 mL of 0.03% Tween 80 and agitated on a vortex mixer for 2 min to dislodge conidia. Each suspension was then diluted 100‐fold and spread onto 10 mL of SDA in a 90 mm Petri dish. This was repeated after each bioassay treatment, totalling three replicates per isolate. Dishes were incubated at 25 °C in the dark for 5 days before CFUs were counted and dose of spray received (conidia mm^–2^) was calculated.

### 2.5 Statistical analysis

Data to assess the relationship between the volume of spray applied and the volume received in the target spray area at a range of spray pressures were subjected to analysis of variance (ANOVA) followed by Tukey's range test. Deposition of green dye on each cover slip after 1 mL application to assess differential deposition of spray across target spray areas was normalised using a log10 transformation and subjected to an ANOVA followed by Tukey's range test. Similarly, data for conidia deposition after application of a range of *B. bassiana* isolate PPRI5339 concentrations were transformed using a natural logarithm. Differences in the number of conidia received on each cover slip in each replicated experiment were determined by conducting ANOVAs. This data was modelled as a linear regression as well as a second‐, third‐ and fourth‐order polynomial regression and the fit of each model was compared using ANOVAs. The relationship between nymph dispersion and the number of nymphs per leaf was analysed using a Pearson's correlation coefficient.

Dose and total proportion mortality at the end of the bioassay data could not be normalised and so were analysed using the Kruskal–Wallis test followed by a *post hoc* Dunn test. Control mortality was corrected for using Schneider–Orelli's formula,^42^ where corrected mortality (%) = (*a* – *b*/100 – *b*) × 100 (where *a* = percentage mortality data from the treated group and *b* = percentage mortality from the control group). The relationship between dose received in each treatment and total nymph mortality was assessed using a Pearson correlation. A non‐parametric survival analysis was conducted by creating Kaplan–Meier curves for each treatment. Differences in mortality caused by each treatment were analysed using a log‐rank test, followed by multiple pairwise log‐rank comparisons. Multiple pairwise comparisons of isolates causes an increase in overall type 1 error, so Bonferroni adjustments were applied to the *P* value. A Cox proportional hazards model was created to estimate the mean increase in risk of mortality of *T. vaporariorum* in treated groups compared to the control.

All analyses were conducted in R (version 1.1.419). The survival package was used to conduct the Kaplan–Meier curves and the Cox proportional hazard model.^43^

## 3 RESULTS

### 3.1 Sprayer calibration

The volume of water deposited across each 90 mm Petri dish was significantly different depending on the application pressure, which ranged from 103 to 207 kPa (df = 3, *F* = 5.3, *P* < 0.001) as shown in Table 2. Average deposition decreased with increasing pressure except at 207 kPa, which could have been caused by the spray gun ‘spitting’ instead of producing a uniform mist at the highest pressure setting. However, only deposition rates at 103 and 172 kPa were significantly different (*t* value = 12.66, *P* = 0.007; Table 2). Visual inspection of water droplets on Petri dish lids indicated that a higher volume of water was received in the central area compared to the outer edge of each dish and water droplet size increased with application pressure above 137 kPa (Fig. 1(b)).

**Table 2:** Volume of liquid deposited on 90 mm Petri dish lids after being sprayed with 1 mL of deionised water at pressures ranging from 103 to 207 kPa and the estimated field application volume that this would represent

Pressure (kPa)	Total volume deposited (mL ± SD)	Volume deposited per unit area (mL mm^−2^)	L/ha^a^
103	0.42 ± 0.018^a^	0.067 ± 0.003	666.93
138	0.39 ±0.021^ab^	0.062 ± 0.003	617.13
172	0.38 ± 0.023^b^	0.059 ± 0.004	590.29
207	0.39 ±0.017^ab^	0.061 ± 0.003	611.63

Means not followed by the same letter are significantly different as indicated by 95% confidence limits.

a Based on 1 ha = 1 × 10^10^ mm^2^.

The volume of green dye applied to each coverslip arranged within the central 45 mm of Petri dishes to assess differential deposition within the spray target area was not significantly different in each replicate experiment (df = 2, χ^2^ = 0.75, *P* = 0.69, Kruskal–Wallis test), therefore data were combined for analysis. The mean volume of green dye received by each coverslip on the Petri dish differed significantly when applied with 172 kPa, giving an average deposition of 114 ± 30 μL mm^−2^ (df = 4, χ^2^ = 30.36, *P* = <0.001). In comparison, coverslip position did not affect the mean volume of green dye received at 103 kPa (130 ± 16 μL mm^−2^; df = 2, *F* = 0.047, *P* = 0.96) or 138 kPa (110 ± 4 μL mm^−2^; df = 2, *F* = 0.48, *P* = 0.622).

**Figure 2:**
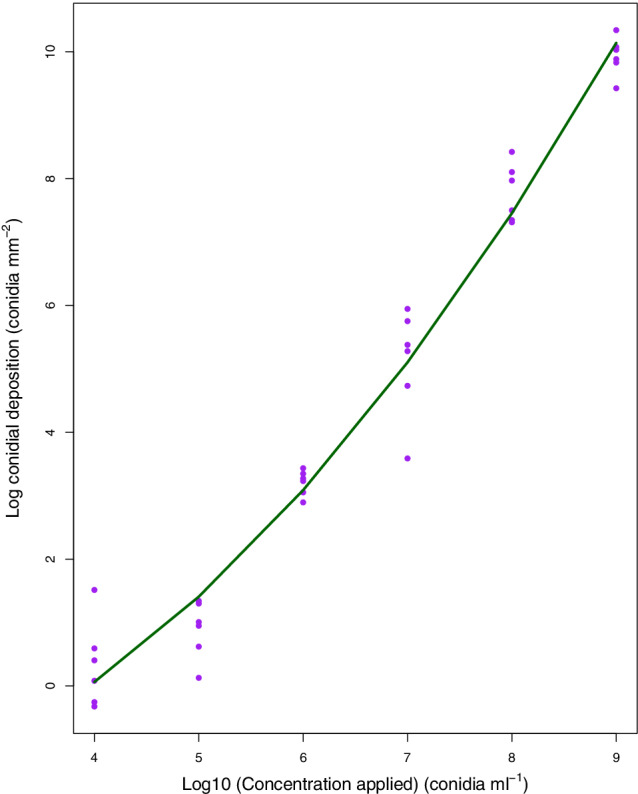
Concentration of *B. bassiana* PPRI5339 conidia deposited on a 22 × 22 mm square coverslip (log conidia mm^−2^), pooled across replicate experiments, after being applied at a range of concentrations and 138 kPa. The regression equation for the fitted curve is log conidia deposition ~ PPRI5339 concentration + PPRI5339 concentration^2^ provided the best fit for the data (ANOVA, *F* = 608.4, df = 33 *P* < 0.001) and explained 97.2% of the variation in the data.

The mean number of *B. bassiana* (PPRI5339) conidia received per 22 × 22 mm coverslip ranged from 2.62 ± 1.68 conidia mm^−2^ at an application concentration of 1 × 10^4^ conidia mL^−1^ to 2.41 × 10^4^ ± 0.63 × 10^4^ conidia mm^−2^ at an application concentration of 1 × 10^9^ conidia mL^−1^. The mean number of conidia mm^−2^ received per coverslip increased with application concentration but the relationship was not linear (Fig. 2). A quadratic model (log conidia deposition ~ PPRI5339 concentration + PPRI5339 concentration^2^) provided the best fit for the data (ANOVA, *F* = 608.4, df = 33 *P* < 0.001, *r* = 0.97; Fig. 2).

### 3.2 Bioassay results

Adult *T. vaporariorum* were restricted to a 15 mm diameter circle (area 177 mm^2^) on the centre of the abaxial leaf surface to ensure eggs were oviposited within this area. The number of eggs oviposited per leaf ranged from a minimum of eight to a maximum of 97, with an average of 40 ± 23 eggs per leaf. On average, 91% of eggs hatched successfully and nymphs were subsequently counted when they reached the third‐instar stage, at which point they were dispersed across an average area of 373.76 ± 5 mm^2^. There was no correlation between number of eggs laid and area of leaf occupied by nymphs (*t* = 1.46, df = 25, *P* = 0.16, Pearson's correlation).

**Figure 3:**
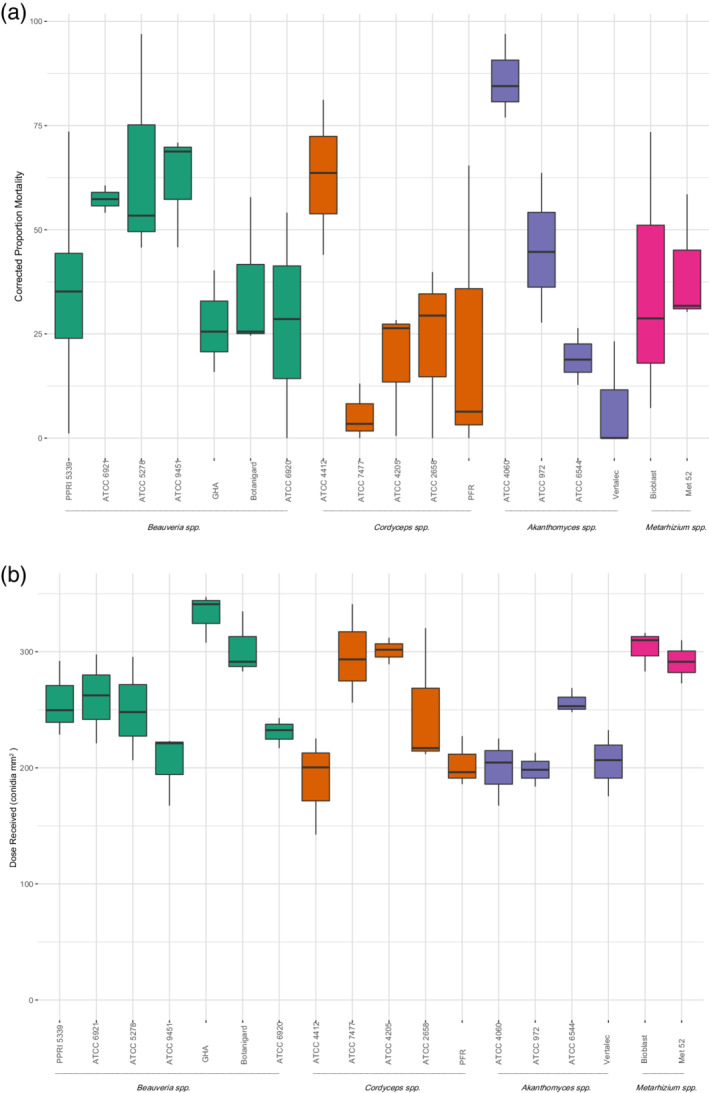
(a.) Average percentage mortality of third‐instar greenhouse whitefly (*T. vaporariorum*) 14 days after application of 1 mL of 1 × 10^7^ conidia mL^−1^ of 18 different entomopathogenic fungal isolates applied at 138 kPa. Control mortality (3%) was corrected for using Schneider–Orelli's formula: corrected mortality (%) = (*a* – *b*/100 – *b*) × 100, where *a* is the percentage mortality data from the treated group and *b* is the percentage mortality from control group. (b) Dose received per mm^2^ of 22 × 22 mm coverslips in the centre of a Petri dish sprayed with 1 mL of 1 × 10^7^ conidia mL^−1^ of 18 different entomopathogenic fungal isolates. Error bars indicate standard deviation.

Throughout the bioassay experiments, *B. bassiana* isolate PPRI5339 was included as a standard in each of the three groups to compare consistency between experiments. Conidial deposition for *B. bassiana* isolate PPRI5339 ranged from 213 to 320 conidia mm^−2^ and was not significantly different between the groups (df = 2, *F* = 1.903, *P* = 0.24), therefore data across all the assays were combined in a single analysis. Conidial deposition varied for each isolate (df = 19, *F* = 4.93, *P* = <0.001) and ranged from 189 to 332 conidia mm^−2^, equivalent to 1.9 to 3.3 × 10^12^ conidia ha^−1^ (Table 3). Conidial deposition was significantly different when isolates were grouped by genus (df = 3, *F* = 4.098, *P* = 0.01). A *post hoc* Tukey showed that conidial deposition for *Beauveria* spp. was not significantly different from *Cordyceps* spp. (*P* = 0.70) and *Metarhizium* spp. (*P* = 0.16). However, the average number of *Akanthomyces* spp. conidia deposited was significantly lower than for *Metarhizium* spp. isolates (*P* = 0.008; Table 4). Total proportion mortality caused by the replicated PPRI5339 isolate in each group did not differ significantly (df = 2, *F* = 0.82, *P* = 0.49), so bioassay mortality results were combined in a single analysis. Despite small differences in dose received by target *T. vaporariorum* (Fig. 3), there was no correlation between dose received and total proportion mortality observed for all *Beauveria* spp. (df = 23, *t* = −0.48, *P* = 0.64), *Cordyceps* spp. (df = 13, *t* = −1.4, *P* = 0.17), *Akanthomyces* spp. (df = 9, *t* = −1.0, *P* = 0.35) or *Metarhizium* spp. isolates (df = 4, *t* = 0.39, *P* = 0.72).

**Table 3:** Average conidial deposition after 1 mL of 1 × 10^7^ conidia mL^−1^ of 18 different EPF was sprayed onto a 22 × 22 mm square coverslip using the spray tower at 137.9 kPa

ARSEF ID/name	Species	Deposition (conidia mm^2^) mean ± SE	Equivalent field deposition (conidia ha^−1^)^a^
ATCC 5278	*Beauveria bassiana*	250 ± 44.46	2.5 × 10^12^
ATCC 6920	*Beauveria bassiana*	231 ± 12.99	2.3 × 10^12^
ATCC 6921	*Beauveria bassiana*	260 ± 38.30	2.6 × 10^12^
ATCC 9451	*Beauveria bassiana*	204 ± 31.63	2.04 × 10^12^
Botanigard	*Beauveria bassiana*	303 ± 27.75	3.03 × 10^12^
GHA	*Beauveria bassiana*	332 ± 21.10	3.32 × 10^12^
PPRI5339	*Beauveria bassiana*	257 ± 44.38	2.57 × 10^12^
ATCC 7477	*Cordyceps javanica*	297 ± 42.46	2.97 × 10^12^
ATCC 4412	*Cordyceps farinosa*	189 ± 42.41	1.89 × 10^12^
ATCC 2658	*Cordyceps fumosorosea*	250 ± 61.19	2.49 × 10^12^
PFR	*Cordyceps fumosorosea*	203 ± 21.50	2.03 × 10^12^
ATCC 4205	*Cordyceps fumosorosea*	301 ± 11.38	3.01 × 10^12^
ATCC 4060	*Akanthomyces lecanii*	199 ± 29.32	1.99 × 10^12^
ATCC 972	*Akanthomyces lecanii*	198 ± 35.31	1.98 × 10^12^
ATCC 6544	*Akanthomyces lecanii*	257 ± 10.75	2.57 × 10^12^
Vertelec	*Akanthomyces muscarius*	205 ± 28.45	2.05 × 10^12^
Met 52	*Metarhizium brunneum*	291 ± 18.60	2.91 × 10^12^
Bioblast	*Metarhizium anisopliae*	303 ± 17.57	3.03 × 10^12^

a Field deposition calculated based on 1 ha = 1 × 10^10^ mm^2^.

**Table 4:** Average dose received after 1 mL of 1 × 10^7^ conidia mL^−1^ of 18 different EPF were sprayed onto a 22 × 22mm square coverslip using the spray tower

Genus	Average dose received after spray application (conidia mm^−2^)
*Beauveria* spp.	262 ± 49^ab^
*Cordyceps* spp.	248 ± 58^ac^
*Akanthomyces* spp.	216 ± 33^c^
*Metarhizium* spp.	297 ± 17^b^

Means not followed by the same letter are significantly different as indicated by 95% confidence limits.

**Figure 4:**
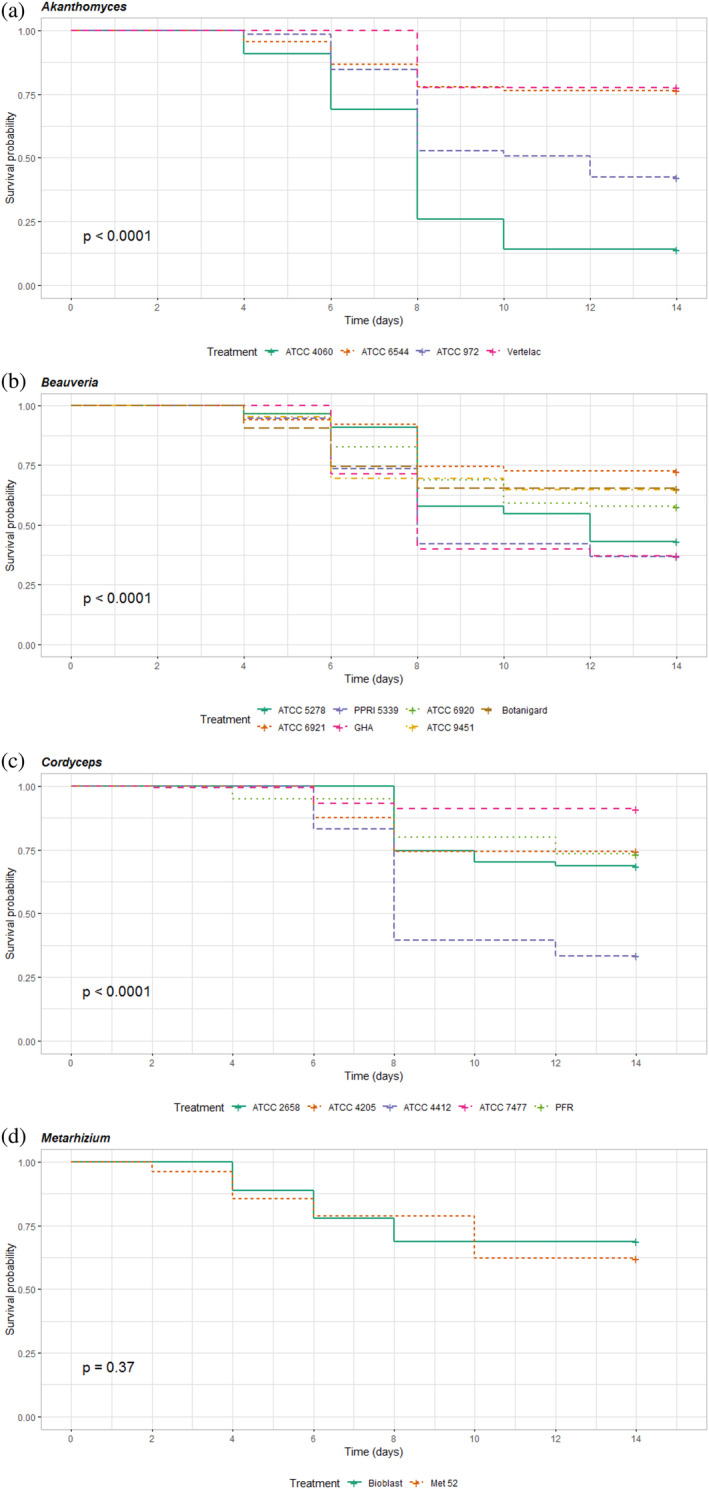
Kaplan–Meier survival curves for *T. vaporariorum* treated with 1 × 10^7^ conidia mL^−1^ of 18 different EPF, separated by genus into (a) *Akanthomyces* isolates, (b) *Beauveria *isolates, (c) *Cordyceps* isolates and (d) *Metarhizium* isolates. *P* values indicate differences in survival rate 14 days after *T. vaporariorum* were exposed to different isolates of the same genus using log rank tests for overall comparison.

Fungal‐induced mortality and control mortality (non‐fungal) were observed as early as 2 days after leaves were sprayed with all treatments. All EPF applied were pathogenic to *T. vaporariorum*, as shown by total proportion mortality (Fig. 3) and Kaplan–Meier survival curves (Fig. 4), which differed significantly from the control, irrespective of the species or strain applied (Kaplan–Meier log rank: df = 18, χ^2^ = 437, *P* < 0.001; Kruskal–Wallis test df = 18, χ^2^ = 44.50, *P* < 0.001). After 14 days, total mortality ranged between 8.1 ± 7.3% and 89 ± 10% depending on the isolate applied. The average mortality in the control group (treated with 0.03% Tween 80) was 3.2 ± 6.3%, with all surviving nymphs emerging as adults. Isolate 4060 of *A. lecanii*, 9451, 5278 and 6921 of *B. bassiana*, and 4412 of *C. farinosa* were more pathogenic to *T. vaporariorum* nymphs (≥60%) than all other isolates and overall isolate 4060 caused the greatest mortality in *T. vaporariorum* nymphs (89%), which was >50 times higher than control mortality (Cox proportional hazards: 95% CI: 27.6–118). The Kaplan–Meier curve estimated for this isolate was significantly different to all other isolates except *C. farinosa* 4412 (*P* = 0.053), *B. bassiana* 6921 (*P* = 1.00) and *B. bassiana* 9451 (*P* = 0.21).

The Kaplan–Meier survival analysis indicated there were significant differences in mortality caused by isolates within the genera *Beauveria* (*P* < 0.0001), *Cordyceps* (*C. farinosa*, *C. fumosorosea*, *C. javanica*) (*P* < 0.0001) and *Akanthomyces* (*P* < 0.0001) but there was no significant difference in survival of *T. vaporariorum* when treated with *M. brunneum* (Met 52) or *M. anisopliae* (Bioblast) (*P* = 0.37), as shown in Fig. 4.

## 4 DISCUSSION

Here, we present a standardised bioassay method using a calibrated laboratory spray tower and novel clip cage design to deliver repeatable doses of EPF for simple assessment of the pathogenicity and relative virulence of fungal entomopathogens against whitefly. The sprayer delivered a uniform spray volume to the central 1590 mm^2^ of the spray area. Restricting *T. vaporariorum* adults to a single leaf using the clip cage allowed egg laying and subsequent developing nymphs to be successfully contained in a known area to ensure they received a uniform spray coverage. By introducing a known number of adults, there was an added advantage that the cages allowed some control over the resultant replicate sample sizes. Several clip cages have been developed in the past, many of them following the same basic design by MacGillivray and Anderson^41^ with some varying in shape to allow for *T. vaporariorum* natural behaviours.^44^ First‐instar *T. vaporariorum* crawl across the leaf surface before securing themselves at a preferred feeding site where they remain until emerging as adults and there is a lack of information on the ability of clip cages to confine *T. vaporariorum* to a known area once the cage and *T. vaporariorum* adults have been removed. In the current study, *T. vaporariorum* nymphs migrated to cover a maximum area of 380 mm^2^ but never onto the upper leaf surface. By restricting egg laying to 177 mm^2^, the distribution of third instars was within the central 1590 mm^2^ which received uniform coverage at 138 kPa.

The applied volume, size of droplets and uniformity across the spraying area are affected by the pressure used during application of pesticides. In comparison to work by Mascarin *et al*.,^20^ our assays applied a known volume during both calibration and subsequent bioassays instead of spraying based on time (seconds), which can introduce variation due to human error, as found in preliminary observations. Spray pressure at 138 kPa was selected as the preferred setting based on the percentage volume reaching the target and uniformity of spray across the target area. As application pressure was increased, the volume of liquid reaching the target area decreased, similar to findings by Liu and Stansly^45^ when using the Potter spray tower. However, unlike the calibration of the Potter spray tower, the spray received was more uniform at lower application pressures. This was likely due to the spray gun spitting at higher pressures, resulting in uneven application of the solution. The portable spray tower designed by Mascarin *et al*.^39^ delivered 150 ± 34 conidia mm^−2^ following an application of 4 mL of 1 × 10^7^ conidia mL^−1^ of three different EPF at 69 kPa. In comparison, our tower delivered an average of 256 ± 31 conidia mm^−2^ after a 1 mL application of 1 × 10^7^ conidia mL^−1^ of 18 different fungal isolates. This variation demonstrates the importance of calibration of sprayers under different laboratory conditions.

Previously reported EPF bioassays against *T. vaporariorum* have most often used the leaf disc method^20^, ^46^, ^47^, ^48^ or detached leaves^20^ to expose whitefly to fungal conidia. Nutritional reserves of detached leaves are limited and the duration of these bioassays can be constrained due to plant senescence. Variation in plant quality plays a role in insect development and growth, causing variation in susceptibility of insects to infection by EPF.^49^ The amount and type of plant volatiles produced in response to damage are different depending on whether detached leaves or whole plants are used.^50^ Therefore, results from bioassays using detached or cut leaves should be cautiously interpreted. By using healthy whole plants, changes at the microclimatic scale are reduced and bioassay results are less likely to be altered by plant surface chemistry.^51^

The leaf‐dip bioassay is also a common method used to test the efficacy of pesticides in laboratory bioassays with sucking insects that are not easily removed from the leaf. This method ensures that every target nymph is exposed to the insecticide and is useful as a quick and cheap screening process for bioprotectants. However, it can be difficult to determine dose due to variation in duration of immersion and the gradual removal of conidia when multiple leaves are sequentially dipped into a stock suspension.^52^ Mortality caused by surfactants such as Tween 80 and Triton‐X has been shown to be greater in immersion/leaf‐dip bioassays compared to spray applications and the potential for nymphs to drown can also be higher following the leaf‐dip method.^53^, ^54^, ^55^ The spray equipment and bioassay method we report here is cheap to assemble and dose can be quantified to ensure that variation is minimal, unlike the leaf‐dip bioassay.

In this study, the spray tower was used to apply EPF onto target *T. vaporariorum* nymphs in pathogenicity bioassays at a single concentration of 1 × 10^7^ conidia mL^−1^. *T. vaporariorum* nymphs were susceptible to all EPF isolates tested. However, there was large variation in the total mortality of nymphs, indicating that isolates taken from different hosts and geographical locations have different pathogenicity toward *T. vaporariorum* nymphs. *B. bassiana* isolate GHA originates from *Diabrotica undecimpunctata* and *C. fumosorosea* isolate PFR from Aphididae, yet they infected a higher proportion of whitefly nymphs than some other isolates originating from the target species. Several other studies have found that the most pathogenic isolates may not originate from the target species,^56^, ^57^ which is well known for anamorphic EPF belonging to the order Hypocreales as they generally have an extensive host range,^58^ infecting multiple species across several taxonomic orders.^59^ This characteristic is expected from EPF isolated from commercial products because one of the selection criteria for microbial control agents is high virulence toward a broad range of pest species, amongst other traits such as their ability to be mass produced on artificial media. Of the 18 fungal isolates used in bioassays against *T. vaporariorum*, *A.lecanii* isolate 4060 caused the highest overall mortality. Although this is only at a single‐dose application to estimate pathogenicity, and it must be noted that over a range of doses the LD_50_ values for isolates would most likely rank isolates in a different manner, the tests described in this study can be used to validate the effectiveness of different EPF against *T. vaporariorum*.

Mortality caused by EPF is also affected by methods used to handle and apply the pathogen.^60^ The number of conidia reaching the target nymphs in the single‐dose response bioassay ranged from 189 to 332 conidia mm^−2^. There was no significant effect of conidial deposition on *T. vaporariorum* mortality, but application of *Akanthomyces* isolates resulted in significantly fewer conidia deposited onto the target area than *Metarhizium* isolates. However, there was no significant difference in conidial deposition of *Akanthomyces* isolates and *Beauveria* or *Cordyceps* isolates, therefore these differences are unlikely to have been caused by differences in conidia suspensions caused by the hydrophobicity of *Beauveria*, *Cordyceps* and *Metarhizium* conidia, compared to the hydrophilic conidia of *Akanthomyces* isolates. Instead, we suggest that the small variation in conidia number could be attributed to counting errors in the haemocytometer and potentially small differences in percentage germination of conidia from different species. Furthermore, our study demonstrated an effective way to quantify dose and ensure conidial viability by counting CFUs after conidia were sprayed through the spray tower, which will account for the variation in percentage germination of conidia through differences in the number of CFUs.

Inundative control of whitefly using EPF in greenhouse or field crops requires at least 10^12^–10^14^ conidia per hectare.^58^, ^61^ Conidia received per unit area using the calibrated spray tower are equivalent to the concentration applied to field crops and therefore mortality results from this laboratory might be more representative of field efficacy than leaf‐dip bioassays, although spray cover in the field will be more patchy due to leaf shielding and efficacy is strongly influenced by prevailing environmental conditions.

The standardised bioassay method we have described could be easily adapted to test the virulence of EPF in dose‐response bioassays. Increasing the application concentration of *B. bassiana* isolate PPRI5339 resulted in an increased deposition of conidia to the target area. This was a non‐linear relationship which may be due to conidia being sprayed onto the sides of the spray tower during application. By calibrating the spray tower and measuring the dose received by the known target area, LD_50_ and LC_50_ could be calculated and virulence against *T. vaporariorum* could be directly compared in the future. The spray tower is equally capable of uniformly applying synthetic chemical insecticides and 100% mortality of *T. vaporariorum* nymphs was observed when recommended application rates of Admiral Advance (active ingredient: pyriproxyfen) were applied in preliminary tests (Spence, unpublished data). Investigations to improve understanding of EPF interactions with other pesticides could be conducted using this bioassay protocol.

The successful development of EPF for control of *T. vaporariorum* relies, in part, on the initial selection of the most efficacious isolates or strains of different species. The laboratory bioassay method we describe in this study provided an efficient, repeatable assay for determining the pathogenicity of 18 isolates from four genera of fungi and could be further extended to determine virulence across different doses of fungi, prior to larger scale mesocosm or glasshouse trials. This assay approach, using an affordable, small‐scale and easily replicated spray tower design, provides a useful method for simple assessment of EPF for control of *T. vaporariorum* and has the potential to be extended to other phloem‐feeding pests.

### ACKOWLEDGEMENTS

This study was funded by a NERC CASE studentship award granted to the Centre for Ecology & Hydrology (NERC reference: NE/P010490/1) and the University of Warwick. Additional funding and support as part of the award is provided by BASF and CAB International. We thank Philip Farrand and team (Centre for Ecology & Hydrology, Wallingford) for constructing the spray towers.
